# Prevalence and Determinants of Depressive and Anxiety Symptoms among Transgender People: Results of a Survey

**DOI:** 10.3390/healthcare11050705

**Published:** 2023-02-27

**Authors:** André Hajek, Hans-Helmut König, Elzbieta Buczak-Stec, Marco Blessmann, Katharina Grupp

**Affiliations:** 1 Department of Health Economics and Health Services Research, University Medical Center Hamburg-Eppendorf, Hamburg Center for Health Economics, 20246 Hamburg, Germany; 2 Division of Plastic, Reconstructive and Aesthetic Surgery, University Medical Center Hamburg-Eppendorf, 20246 Hamburg, Germany

**Keywords:** mental health, depression, anxiety, transgender, transgender people, transgender identity, gender minority adults, gender identity

## Abstract

Objectives: The aim was to investigate the prevalence of probable depression and probable anxiety and to investigate the determinants of depressive symptoms and anxiety symptoms among transgender people. Methods: In this “Transgender Survey” (n = 104) we included transgender people who had joined self-help groups to obtain and share information about the gender-affirming surgeries performed at the Division of Plastic, Reconstructive and Aesthetic Surgery at the University Medical Center Hamburg-Eppendorf. Data collection took place between April and October 2022. To measure probable depression, the patient health questionnaire-9 was used. The generalized anxiety disorder-7 was used to quantify probable anxiety. Results: The prevalence of probable depression was 33.3% and it was 29.6% for probable anxiety. Multiple linear regressions showed that both more depressive symptoms and anxiety symptoms were significantly associated with younger age (β = −0.16, *p* < 0.01; β = −0.14, *p* < 0.01), being unemployed (e.g., full-time employed compared to unemployment: β = −3.05, *p* < 0.05; β = −2.69, *p* < 0.05), worse self-rated health (β = −3.31, *p* < 0.001; β = −1.88, *p* < 0.05), and having at least one chronic disease (β = 3.71, *p* < 0.01; β = 2.61, *p* < 0.05). Conclusions: Remarkably high prevalence rates were identified among transgender people. Furthermore, risk factors of poor mental health (e.g., unemployment or younger age) were identified—which can help to address transgender people at risk for poor mental health.

## 1. Introduction

Worldwide, 264 million people (3.6%) suffer from an anxiety disorder and 322 million (4.4%) from a depressive disorder [1]. Thus, depression and anxiety are the most common mental disorders in the general population [2].

Transgender people identify with a different gender from their sex assigned at birth [3]. Trans feminine describes transgender people assigned a male sex at birth who identify as female and trans masculine describes transgender people assigned a female sex at birth who identify as men. Transgender people have diverse sexual orientation identities, attractions, and behaviors. Globally, accurate data concerning the size of the transgender population are lacking and population prevalence depends on the definition of transgender [4]. It is estimated that the prevalence of transgender identity ranges between 0.3% and 0.5% [4].

The gender affirmation, which is defined as a process through which a person’s gender identity is socially recognized, is of high importance for transgender people’s health [5].

For transgender people, health inequities are multifactorial and include increased risks for systematic social and economic marginalization, stigma, and discrimination [1]. Transgender people are at substantially increased risk for various negative mental health outcomes compared to cisgender people [3]. These poor mental health outcomes include depression and anxiety [6,7,8,9,10] and are likely due to social rejection [11,12], stigma, discrimination, and violence [13]. 

Moreover, transgender people face difficulties in receiving the diagnosis and obtaining care due to, for example, barriers to mental health care or/and experiencing stigma and discrimination by healthcare professionals or/and lack of support by their families [14]. For example, transgender people often find in healthcare that providers lack skills in the area and are confronted with discrimination. Consequently, providers are often seen as unsupportive to transgender people’s healthcare needs [15].

In summary, for transgender people, healthcare is often inadequate and does not meet the needs of their special requirements [16]. Moreover, studies have shown that lack of support by their families is linked to higher levels of depressive symptoms in transgender people [17].

Negative mental health outcomes, such as suicide or substance use are also associated with increased levels of depression and anxiety. Previously, studies have shown that substance use has been described as a coping mechanism of transgender people to manage enhanced levels of stress [18] and that gender incongruent individuals reported higher rates of life time suicidal ideation and life time suicide attempts (about 61% and 31%) than their cisgender peers (about 20% and 7%) [19].

Earlier studies of depression and anxiety used diverse clinical screening cut-points for clinical syndromes, differing timeframes of assessment, and heterogeneous subpopulations of transgender people. However, high rates of negative mental health outcomes have been reported. For example, depression prevalence estimates are as high as 64.2% in 573 [20] and 63.0% in 230 transgender people using Center for Epidemiologic Studies Depression scale [21], while other authors using clinical diagnosis of depression reported depression rates of 31.4% in 207 [22] and 36.2% in 253 transgender people [23]. Moreover, Bockting et al. [24] and Grant et al. [25] found a prevalence of 44% for depression and 33% for anxiety symptoms.

Earlier studies have suggested that there are potential risk factors influencing the negative mental health outcomes for depression and anxiety. For example, puberty blockers, gender-affirming hormones and gender-affirming surgeries have a relevant clinical impact on mental health and decrease rates of adverse mental health outcomes, such as depression and anxiety in transgender people [22,26,27,28]. Puberty blockers and gender-affirming hormones were linked to improved psychological functioning [29] and decreased depressive symptoms [30]. Additionally, the development of mental health problems has been suggested to be correlated with social connectedness [31,32,33,34], academic performance [35], financial aspects [36,37,38], and chronic illnesses [36]. 

Given the high risk of depression, anxiety, and suicidality among transgender people, there is a pressing need to better characterize mental health and its influencing factors. In light of the restricted knowledge in this area (e.g., studies exclusively focusing on transgender persons and exploring the correlates of mental health in such groups), the aim of our study was to investigate the prevalence of probable depression and probable anxiety and to investigate the determinants of depressive symptoms and anxiety symptoms among transgender people. 

In this study, we demonstrate that depression was detected in 33.3% and that anxiety was found in 29.6% of transgender people. Moreover, we show that school education and having chronic diseases were significantly associated with depression and anxiety. Regressions analyses show that depressive and anxiety symptoms were positively significantly associated with younger age, being unemployed, having worse self-rated health, and having at least one chronic disease. These data reveal new aspects regarding the correlates of mental health in transgender people.

## 2. Materials and Methods

### 2.1. Sample

The Division for Plastic, Reconstructive and Esthetic Surgery and the Department of Health Economics and Health Services Research (both at the University Medical Center Hamburg-Eppendorf, UKE) collaborated on the “Transgender Survey”. Data were taken from this study. 

Regarding inclusion: In this “Transgender Survey” (n = 104) HH-TPCHIVG, we included transgender people who had joined self-help groups (e.g., Facebook, Whatsapp) to obtain and share information about the gender-affirming surgeries performed at the Division of Plastic, Reconstructive and Aesthetic Surgery at the University Medical Center Hamburg-Eppendorf. Included were transgender people before and after gender affirmation surgery at the Division of Plastic, Reconstructive and Aesthetic Surgery at the University Medical Center Hamburg-Eppendorf. The description “transgender people” was accepted by all participants in the study. No particular exclusion criteria, such as those related to age, were used. We programmed, hosted, and carried out the survey using the online survey application “Limesurvey”. Data were gathered between April and October 2022. 

All of the study’s participants provided their informed consent. The study was approved by the Local Psychological Ethics Committee of the Center for Psychosocial Medicine of the University Medical Center Hamburg-Eppendorf (number: LPEK-0480).

### 2.2. Dependent Variables

The established patient health questionnaire-9 (PHQ-9) was used to assess probable depression [39]. It has nine items. Based on these nine items, a sum score can be computed, which ranges from 0 to 27, with higher values reflecting more depressive symptoms. Following the recommendations of prior research [40], we used a PHQ-9 score of ten or higher as the cut-off for probable depression. In this study, Cronbach’s alpha was 0.91 (McDonald’s omega was 0.91).

The established generalized anxiety disorder-7 (GAD-7) [41] was used to assess probable anxiety. The scale has seven items. Based on these items, a sum score was created (from 0 to 21, higher values correspond to more anxiety symptoms). In accordance with the given recommendations [41], we used a cut-off score of ten or higher for probable anxiety. Cronbach’s alpha was 0.91 in our study (McDonald’s omega was 0.91). 

### 2.3. Determinants

According to prior research [42,43,44], we selected these sociodemographic, lifestyle- and health-related determinants: age in years, family situation (five categories: widowed; single; divorced; living separately: married or in partnership; living together: married or in partnership), highest school education (general or subject-specific university entrance qualification (e.g., “Abitur”); completion of polytechnic secondary school; currently in school education; secondary school diploma; intermediate school leaving certificate (e.g., “Realschulabschluss”); without general school leaving certificate), labor force participation (full-time employed; unemployed; part-time employed; marginally employed (450-euro job or mini-job); retired/early retirement; other; in retraining; in vocational training/apprenticeship), migration background (no or yes), religious affiliation (non-denominational; Buddhism; Christianity; Islam; other), already having had gender reassignment surgery (no or yes), frequency of sports activities (no sports activity; less than one hour a week; regularly, 1–2 h a week; regularly 3–4 h a week; regularly, more than 4 h a week), having one or more chronic diseases (no or yes), and self-rated health (single-item, ranging from 1 (very bad) to 5 (very good)). 

### 2.4. Statistical Analysis

The prevalence of probable depression and probable anxiety among transgender people was first displayed. We also displayed the prevalence rates for important subgroups (age bracket, family situation, school education, migration background, labor force participation, having a religious affiliation, already having had gender assignment surgery, and chronic diseases). Additionally, the determinants of depressive symptoms and anxiety symptoms were investigated based on multiple linear regressions. A full-information maximum likelihood (FIML) approach [45] was used to tackle missing values. To calculate McDonald’s omega, a tool was used which was recently developed by Shaw [46]. Statistical significance was defined as *p* < 0.05. Stata 16.1 (Stata Corp., College Station, TX, USA) was used to conduct the statistical analyses.

## 3. Results

### 3.1. Sample Characteristics

In our sample (n = 104), the average age was 30.4 years (SD: 9.6 years), ranging from 19 to 63 years. The prevalence rates are displayed in Table 1. The results are stratified by age group, family situation, school education, migration background, labor force participation, having a religious affiliation, already having had gender assignment surgery, and chronic diseases. The prevalence of probable depression was 33.3% and it was 29.6% for probable anxiety in the total sample. Moreover, 21.4% of transgender people had both probable depression and probable anxiety. In the subgroups, the prevalence rates of probable depression ranged from 10.2% (among transgender people without chronic diseases) to 56.0% (among transgender people with at least one chronic disease). Moreover, the prevalence rates of probable anxiety ranged from 12.5% (among transgender people without chronic diseases) to 54.5% (among transgender people having a migration background). School education and having chronic diseases were significantly associated with both probable depression and probable anxiety. Please see Table 1 for further details.

### 3.2. Regression Analysis

Results of the multiple linear regression analysis with depressive symptoms and anxiety symptoms as outcome measures are shown in Table 2. With depressive symptoms as the outcome measure, R^2^ was 0.60 and with anxiety symptoms as the outcome measure, R^2^ equaled 0.42. Following the variance inflation factors (VIFs; mean VIF was 1.60 and highest VIF was 2.52), multicollinearity may not be a challenge. Regressions showed that among transgender people, both more depressive symptoms and more anxiety symptoms were significantly associated with younger age (β = −0.16, *p* < 0.01; β = −0.14, *p* < 0.01), being unemployed (e.g., full-time employed compared to unemployment: β = −3.05, *p* < 0.05; β = −2.69, *p* < 0.05), worse self-rated health (β = −3.31, *p* < 0.001; β = −1.88, *p* < 0.05) and having at least one chronic disease (β = 3.71, *p* < 0.01; β = 2.61, *p* < 0.05). 

## 4. Discussion

Transgender people, in comparison to cisgender people, are disproportionately burdened by poor mental health outcomes [47,48]. In this study, we demonstrate that among transgender people, the prevalence of probable depression is 33.3% and anxiety is 29.6%. Additionally, our data reveal that both increased depressive and anxiety symptoms are significantly linked to younger age, being unemployed, worse self-rated health, and having at least one chronic disease.

When interpreting our prevalence rates, it is important to keep in mind that we focused on transgender persons who have joined a self-help group seeking gender-affirming surgery. This may imply that their mental health is better compared to transgender persons not involved in such groups because their internalized stigma or ambivalence may be lower. Thus, future research, ideally based on representative samples, are desirable to confirm our present findings.

Our detected rate of depression (33.3%) is within the range of 28% to 64% reported in other studies of depression among transgender people [20,21,23,24,49,50,51]; see also: [25]. In general, these rates are much higher than the rate of depression and anxiety of their heterosexual counterparts [47]. In detail, Konrad et al. [47] analyzed a total of 535 transgender people, 535 non-transsexual women, and 535 non-transsexual men, and showed that depression was documented in 20% of transgender people versus 7.7% of non-transsexual women and 5.5% of non-transsexual men. Moreover, this study identified anxiety disorders in 5.8% of transgender people versus 1.9% of non-transsexual women and 1.6% of non-transsexual men [47]. 

These results may be due to the experience of increased stress due to the permanent confrontation with stigmatization. In general, transgender people are viewed as unnatural and sexually unnatural, resulting in enhanced poor mental health [52]. Several studies showed that social rejection, lack of support from parents [11,12], and bullying [11,53,54], abuse, discrimination [55], and violence [53] are drivers for poor mental health and well-being. 

For example, family affirmation has been detected as a protective factor against depression and suicide among transgender youth, since it increases the levels of self-esteem and support networks and decreases depression and suicide levels [56,57]. Research in transgenderism has shown that transgender people have less social support than their cisgender counterparts [58]. Regarding transgender groups, social support is associated with better physical health, smaller likelihood of discomfort, and lower scores of depressive symptoms and stress [59].

Moreover, adolescents and transgender youth are especially exposed to situations with risk of violence [60]. Ryan et al. [60] showed that transgender youth are more frequently victims of discrimination, as a result of actual or perceived sexual orientation than adults with high rates of victimization, particularly in school and community settings. Beyond violence, transgender youth face increased risks of violence and bullying in school [54]. Clark et al. [54] performed a nationally representative survey including more than 8000 students describing that more than half of transgender people were afraid someone at school would hurt or bother them, and nearly one in five transgender students reported experiencing bullying at school on a weekly (or more frequent) basis. Relative to their non-transgender peers, transgender youth had increased health and well-being needs [54]. In particular, approximately 40% of transgender people had significant depressive symptoms, had harmed themselves, and had been unable to access health care when they needed it [54]. Moreover, about 20% of transgender students had attempted suicide in the previous 12 months [54].

Furthermore, transgender youth are often confronted with intolerance at home or school, and drop out of education or leave home [16]. Moreover, at work, transgender people face increased levels of discrimination resulting in enhanced rates of unemployment [16]. Reduced education status and being unemployed force transgender people to live on the margins of society [16]. In consequence, there is an increased risk that transgender people face situations associated with unsafe sexual practices and substance abuse, resulting in risk of further ill-health and wellbeing. 

In the general population, the rate of unemployment and youth are known factors which are associated with increased levels of negative mental health outcomes, including depression and anxiety. However, we have to highlight that the transgender population is characterized by even higher rates of unemployment than the general population which may result in even higher rates of depression in this subpopulation. Moreover, transgender youth face even higher levels of stress than the general population, which may result in even higher rates of depression and anxiety.

The majority of studies analyzing mental health among transgender people have analyzed adolescents and adults and reported higher rates of anxiety, depression, and suicidality in comparison to heterosexual people [61,62,63,64]. Interestingly, our data indicate an increased rate of depressed younger transgender people in comparison to older ones. This result is in line with the study by Nuttbrock et al. [21] showing that the association of psychological abuse with depression was stronger among younger rather than among older transgender women. In the study by Nuttbrock et al. [21], the association between psychological gender abuse and major depression was approximately three times higher among younger transgender women (aged 19–30 years) than among older transgender women (aged 31–59 years). The reasons for better resilience to psychological but not physical gender abuse in the older cohort are unknown and should be further studied. However, our finding underlines the importance to address the subset of younger transgender people in multidisciplinary gender care. A recent study has shown that among youths who did not initiate puberty blockers or gender-affirming medical interventions, depressive symptoms and suicidality, as compared to those who received the medical intervention, were 2-fold to 3-fold higher [48]. The authors suggested that risks of depression and suicidality might be mitigated with receiving gender-affirming medications in the context of a multidisciplinary care clinic over the relatively short time frame of 1 year [48]. Interestingly, former research examining the effect of hormonal therapy on anxiety symptoms described that they observed no association between the treatment with medication and the anxiety score [65]. A recent cohort study of transgender youth found that all of their anxiety symptoms improved over a longer follow-up of 11 to 18 months; however, a similar to the study by Tordoff et al. [48] did not observe statistically significant improvements in generalized anxiety. This suggests that anxiety symptoms may take longer to improve after the initiation of medical treatment therapy.

Young transgender people receiving hormonal medication are using gender affirming healthcare, which is also called gender reassignment [66], gender-confirming, or gender affirming healthcare [67]. In general, transgender people use gender affirming healthcare for special health needs. In detail, transgender people do not only need help with their hormonal treatment, they also need help regarding, for example, their reproductive health (e.g., gamete storage) or the use of silicon injections.

Previously, social connectedness has been shown to be an important source of psychological wellbeing and a useful instrument in affirming one’s identity for transgender youth [31,32,33]. Studies have indicated that the social support of family and friends is also an important source of wellbeing in transgender people and result in higher levels of this support are associated with the resilience of individuals [34,68,69]. However, although earlier data suggest that a supportive family is of high importance, we were not able to confirm the importance of the family status. This might be due to differences in the patient cohort or study design—or due to a lack of statistical power. Furthermore, prospective multicenter studies are needed to obtain further insight into this relevant aspect.

Interestingly, our study showed an association between the prevalence of at least one chronic disease and increased levels of anxiety and depression. This result is in line with previous studies that have demonstrated that depression is associated with other health problems, such as cardiovascular, metabolic, and lung diseases [70,71,72]. For example, Bisschop et al. [70] included more than 2000 respondents (age 55–85) in their study and showed that lung disease, arthritis, cardiac disease, and cancer were all positively associated with increased depressive symptoms over time.

Interestingly, we performed our survey during the COVID-19 pandemic. Previously, a study has shown that the prevalence of a probable major depressive disorder was 20.0% and the prevalence of a probable generalized anxiety disorder was 13.4% among the general adult German population during the COVID-19 pandemic [43]. This study showed that probable major depressive disorders and probable generalized anxiety disorders were frequent in the general adult population in Germany and that these prevalence rates were higher compared to almost ten years ago [42]. It can be speculated that these higher rates during the pandemic are caused by several challenges, including social distancing and economic uncertainties of individuals [43].

Individuals with chronic diseases are more often chronically depressed. In fact, an earlier study showed that depression is regarded as one of the most common complications of chronic illnesses, with one-third of those with a serious medical condition experiencing depressive symptoms [73]. Additionally, population-based studies have demonstrated that chronic disease is associated with negative mental health problems, such as depression [74]. Thus, evidence is growing that there might be an increased prevalence of depression in individuals with at least one chronic disease [75].

Another aspect analyzed in our study was the association between labor force participation and depressive, as well as anxiety symptoms in the cohort of transgender people. Here, we were able to show that unemployed transgender people have more depressive and anxiety symptoms, as compared to employed individuals. This result is in line with earlier studies describing that depressive symptoms were significantly associated with labor force participation [37]. In detail, individuals with low or insufficient income and unemployment more often develop mental health issues, whereas individuals with higher income are associated with reduced risk of incident mental health problems [24,37].

To improve the health and well-being of transgender people, beyond social transition, the hormonal and surgical treatments are of high importance to positively influence the health of this group [66].

Regarding our strengths and shortcomings, it should be emphasized that this is one of a few studies dealing with the mental health of transgender individuals. It is a vulnerable population that is commonly quite challenging to access (and often subsumed in the group of LGBT). To measure mental health, valid screening tools were used. However, future studies based on a composite international diagnostic interview [76] are required to confirm our present findings. Additionally, several determinants were included in our study and an FIML approach was used to tackle missing data. The cross-sectional design of this study is a shortcoming of this study and thus future longitudinal studies are needed. Moreover, it is difficult to clarify the generalizability of this hospital sample. It should be noted: an a priori power calculation was not performed. Moreover, a response rate could not be calculated since this was an online sample. Additionally, no data were collected regarding substance use/abuse, adverse childhood experiences, or current stigmatization.

In conclusion, remarkably high prevalence rates for both probable depression and probable anxiety were identified among transgender people. Furthermore, risk factors of poor mental health (e.g., unemployment or younger age) were identified—which can help to address individuals at risk of poor mental health. Upcoming studies in this under researched area are strongly recommended.

## Figures and Tables

**Table 1:** Prevalence of probable depression and probable anxiety among several groups.

	n	Probable Depression	*p*-Value	Probable Anxiety	*p*-Value
Total sample	N = 99	33.3%		29.6%	
Age bracket			0.83		0.39
18 to 29 years	N = 53	32.1%		28.3%	
30 years and older	N = 38	34.2%		36.8%	
Family situation			0.09		0.048
Living separately: married or in partnership; divorced; single; widowed	N = 505	40.8%		40.8%	
Living together: Married or in partnership	N = 42	23.8%		21.4%	
School education			<0.01		<0.01
Absence of general or subject-specific university entrance qualification	N = 52	44.2%		44.2%	
Presence of general or subject-specific university entrance qualification	N = 39	17.9%		15.4%	
Migration background			0.10		0.09
No	N = 80	30.0%		28.7%	
Yes	N = 11	54.5%		54.5%	
Labor force participation			0.10		0.18
Unemployed	N = 16	50.0%		43.8%	
Full-time employed	N = 34	20.6%		20.6%	
Other	N = 41	36.6%		36.6%	
Having a religious affiliation			0.04		0.39
Non-denominational	N = 53	24.5%		28.3%	
Having a religious affiliation	N = 38	44.7%		36.8%	
Already having had gender reassignment surgery			0.71		0.77
No	N = 50	35.4%		31.3%	
Yes	N = 38	31.6%		34.2%	
Chronic diseases			<0.001		<0.001
None	N = 52	10.2%		12.5%	
Presence of at least one chronic disease	N = 50	56.0%		46.0%	

Notes: Chi^2^ tests were performed (*p*-values). Probable depression: PHQ-9 score of ten or higher; probable anxiety: GAD-7 score of ten or higher.

**Table 2:** Determinants of depressive symptoms and anxiety symptoms. Findings of the multiple linear regressions.

Independent Variables	Depressive Symptoms	Anxiety Symptoms
Age (in years)	−0.16 ** (0.05)	−0.14 ** (0.05)
Family situation: -Living together: married or in partnership (reference category: other including [living separately: married or in partnership; divorced; single; widowed])	−0.64 (0.95)	−0.53 (0.84)
School education: -General or subject-specific university entrance qualification (e.g., “Abitur”) (reference: lower school education including [completion of polytechnic secondary school; currently in school education; secondary school diploma; intermediate school leaving certificate (e.g., “Realschulabschluss”); without general school leaving certificate])	−1.93 ^+^ (0.99)	−2.08 * (1.03)
Labor force participation: -Full-time employed (reference category: unemployed)	−3.05 * (1.34)	−2.69 * (1.13)
-other including [part-time employed; marginally employed (450-euro job or mini-job); retired/early retirement; other; in retraining; in vocational training/apprenticeship	−4.16 *** (1.15)	−2.69 * (1.28)
Migration background: Yes (reference category: no)	−0.83 (1.47)	−0.09 (1.25)
Religious affiliation: Having a religious affiliation including [Buddhism; Christianity; Islam; other] (reference category: non-denominational)	0.93 (1.03)	0.47 (0.93)
Already having had gender reassignment surgery: -Yes (reference category: no)	0.35 (0.95)	0.73 (0.97)
Frequency of sports activities: -Less than one hour a week (reference category: no sports activity)	−0.68 (1.28)	−0.27 (1.23)
-Regularly, 1–2 h a week	1.05 (1.40)	−0.09 (1.46)
-Regularly, 3–4 h a week	−0.06 (1.38)	−1.29 (1.31)
-Regularly, more than 4 h a week	0.24 (1.54)	0.40 (1.39)
Self-rated health (from 1 = very bad to 5 = very good)	−3.31 *** (0.70)	−1.88 * (0.76)
Having at least one chronic disease: Yes (Reference category: no)	3.71 ** (1.17)	2.61 * (1.31)
Constant	26.91 *** (3.37)	20.24 *** (4.08)
R^2^	0.60	0.42
Observations	104	104

Notes: Unstandardized beta-coefficients are displayed; robust standard errors in parentheses; *** *p* < 0.001, ** *p* < 0.01, * *p* < 0.05, and + *p* < 0.10; FIML was used to tackle missing data.

## Data Availability

The datasets analyzed during the current study are not publicly available due to ethical restrictions involving patient data but are available from the corresponding author upon reasonable request.
